# Impact of cholecystectomy on acute coronary syndrome according to metabolic condition: a nationwide population-based cohort study

**DOI:** 10.1038/s41598-023-33440-4

**Published:** 2023-05-05

**Authors:** Wonjeong Chae, Hee Seung Lee, Jung Hyun Jo, Moon Jae Chung, Seungmin Bang, Seung Woo Park, Si Young Song, Sung-In Jang, Jeong Youp Park

**Affiliations:** 1 Department of Health Policy and Management, Yonsei University Graduate School of Public Health, 50-1 Yonsei-ro, Seodaemun-gu, Seoul, 03722 Republic of Korea; 2 Institute of Health Services Research, Yonsei University, 50-1 Yonsei-ro, Seodaemun-gu, Seoul, 03722 Republic of Korea; 3 Division of Gastroenterology, Department of Internal Medicine, Yonsei University College of Medicine, 50-1 Yonsei-ro, Seodaemun-gu, Seoul, 03722 Republic of Korea; 4 Department of Preventive Medicine and Institute of Health Services Research, Yonsei University College of Medicine, 50-1 Yonsei-ro, Seodaemun-gu, Seoul, 03722 Republic of Korea

**Keywords:** Cancer, Diseases, Endocrinology, Gastroenterology, Oncology, Risk factors

## Abstract

Gallbladder stones (GS) is associated with an increased risk of cardiovascular disease. However, the relationship between cholecystectomy for GS and acute coronary syndrome (ACS) is unknown. We investigated the ACS risk in patients with GS and its association with cholecystectomy. Data from the Korean National Health Insurance Service-National Sample Cohort from 2002 to 2013 was extracted. Overall, 64,370 individuals were selected through a 1:3 propensity score matching. Patients were stratified into two groups for comparison: the gallstone group, GS patients with or without cholecystectomy; and the control group, patients without GS or cholecystectomy. The gallstone group exhibited a higher risk of ACS than the control group (hazard ratio [HR], 1.30; 95% confidence interval [CI] 1.15–1.47; *P* < 0.0001). In the gallstone group, individuals without cholecystectomy had a higher risk of ACS development (HR: 1.35, 95% CI 1.17–1.55, *P* < 0.0001). Patients with GS with diabetes, hypertension, or dyslipidemia, had a higher risk of developing ACS than GS patients without the metabolic diseases (HR: 1.29, *P* < 0.001). The risk did not significantly differ after cholecystectomy compared to those without GS (HR: 1.15, *P* = 0.1924), but without cholecystectomy, the risk of ACS development was significantly higher than control group (1.30, 95% CI 1.13–1.50, *P* = 0.0004). Among patients without above metabolic disorders, cholecystectomy was still associated with increased ACS risk in the gallstone group (HR: 2.93, 95% CI 1.27–6.76, *P* = 0.0116). GS increased the risk of ACS. The effect of cholecystectomy on ACS risk differs according to the presence or absence of metabolic disorders. Thus, the decision to perform cholecystectomy for GS should consider both the ACS risk and the underlying disorders.

## Introduction

GB stones (GS) are a very common benign gall bladder disease^1–3^. GS and acute coronary syndrome (ACS) share a number of common risk factors, and previous studies have found that the incidence of ACS was higher in people with gallstones^4–7^. Zheng et al. analyzed the relationship between a history of GS and the risk of incident ACS in 3 large prospective US cohorts (n = 269,142 participants)^6^. They found that a history of GS was associated with a 23% (15–33%) increase in ACS risk^6^. Olaiya et al. further reported that patients with GS had an elevated risk of ACS (hazards ratio [HR], 1.42; 95% CI 1.28–1.58).^5^ ACS remains the leading cause of death worldwide. Despite substantial improvements in ACS prevention and treatment, the social and economic burden remains significant and continues to increase^8^. Thus, the association between GS and ACS could be a matter of interest in the prevention and management of ACS.

Cholecystectomy is one of the most frequently performed surgical procedures worldwide, and is considered safe in patients with GS. Even though the association between GS and ACS has been reported a number of times, few studies have observed an association between cholecystectomy and the risk of ACS, and their results are contradictory^9,10^. Chen et al. showed that cholecystectomy could decrease the risk of ACS^10^, and Norberto et al. reported that patients undergoing cholecystectomy had an increased prevalence of risk factors for ACS^9,11^. The outcomes of each study could be contentious as differences in baseline characteristics were not considered in this analysis. As there are other risk factors for ACS, such as hypertension, diabetes mellitus, and hyperlipidemia, we believe that the effects of cholecystectomy on ACS should be analyzed according to the underlying medical conditions.

We sought to investigate the association between GS and cholecystectomy and the risk of ACS according to the underlying medical conditions by conducting a nationwide population-based cohort study to explore the association between cholecystectomy and ACS.

## Results

The baseline characteristics of the study population are presented in Table 1. A total of 64,370 individuals (47.8% men and 52.2% women) were selected for the study. Of these, 20.3% (n = 13,069) with GS were classified in the gallstone group, and 79.7% (n = 51,301) in the control group did not have GS. Among the patients with GS, 20.7% (n = 2706) underwent cholecystectomy, while 79.3% (n = 10,363) did not.

**Table 1:** Baseline characteristics of the study population.

	Acute coronary syndrome						
	Total	%	Yes	%	No	%	*P*-value
	64,370		1279	2.0	63,091	98.0	
Gallbladder stone (GS)							< .0001
Yes	13,069	20.3	357	2.7	12,712	97.3	
Cholecystectomy Yes	2706	20.7	103	3.8	2603	96.2	
Cholecystectomy No	10,363	79.3	254	2.5	10,109	97.5	
No	51,301	79.7	922	1.8	50,379	98.2	
Age group (years)							< .0001
20–29	3100	4.8	5	0.2	3095	99.8	
30–39	9035	14.0	33	0.4	9002	99.6	
40–49	12,628	19.6	103	0.8	12,525	99.2	
50–59	14,548	22.6	272	1.9	14,276	98.1	
60–69	12,484	19.4	443	3.5	12,041	96.5	
70–79	9341	14.5	347	3.7	8994	96.3	
80–89	3234	5.0	76	2.4	3158	97.6	
Sex							< .0001
Male	30,778	47.8	688	2.2	30,090	97.8	
Female	33,592	52.2	591	1.8	33,001	98.2	
Regions							0.0072
Capital area	26,850	41.7	482	1.8	26,368	98.2	
Metropolitan area	16,437	25.5	334	2.0	16,103	98.0	
Rural area	21,083	32.8	463	2.2	20,620	97.8	
Income level							0.0008
Low	11,593	18.0	233	2.0	11,360	98.0	
Medium	26,302	40.9	461	1.8	25,841	98.2	
High	26,475	41.1	585	2.2	25,890	97.8	
Occupational status							< .0001
Working	31,593	49.1	551	1.7	31,042	98.3	
Not working	32,777	50.9	728	2.2	32,049	97.8	
Disability							< .0001
Yes	5528	8.6	172	3.1	5356	96.9	
No	58,842	91.4	1107	1.9	57,735	98.1	
Diabetes							< .0001
Yes	17,610	27.4	702	4.0	16,908	96.0	
No	46,760	72.6	577	1.2	46,183	98.8	
Hypertension							< .0001
Yes	27,667	43.0	1109	4.0	26,558	96.0	
No	36,703	57.0	170	0.5	36,533	99.5	
Dyslipidemia							< .0001
Yes	22,748	35.3	863	3.8	21,885	96.2	
No	41,622	64.7	416	1.0	41,206	99.0	
CCI†							< .0001
0	10,452	16.2	43	0.4	10,409	99.6	
1	9428	14.6	96	1.0	9332	99.0	
2	9978	15.5	119	1.2	9859	98.8	
3	34,512	53.6	1021	3.0	33,491	97.0	
Cohort entry year							< .0001
2004	4902	7.6	198	4.0	4704	96.0	
2005	5271	8.2	197	3.7	5074	96.3	
2006	5590	8.7	182	3.3	5408	96.7	
2007	6065	9.4	146	2.4	5919	97.6	
2008	6346	9.9	162	2.6	6184	97.4	
2009	6141	9.5	116	1.9	6025	98.1	
2010	6640	10.3	109	1.6	6531	98.4	
2011	7741	12.0	80	1.0	7661	99.0	
2012	8303	12.9	58	0.7	8245	99.3	
2013	7371	11.5	31	0.4	7340	99.6	

^**†**^Charlson Comorbidity Index.

Among the study population, individuals with GS had a higher prevalence of diabetes (34.5% vs. 25.5%, *P* < 0.0001), hypertension (48.2% vs. 41.6%, *P* < 0.0001), and dyslipidemia (42.9% vs. 33.4%, *P* < 0.0001) than those without GS. Furthermore, in the gallstone group, the prevalence of the following diseases was slightly higher among patients who underwent cholecystectomy: diabetes (36.0% vs. 34.2%, *P* < 0.0001), hypertension (49.7% vs. 47.9%, *P* < 0.0001), and dyslipidemia (43.7% vs. 42.7%, *P* < 0.0001) (Supplementary Table 1).

Of the total study population, 2.0% (n = 1,279) had ACS. The GS group contained 2.7% (n = 357) ACS patients, while the control group contained 1.8% (n = 922). Furthermore, in the gallstone group, 3.8% (n = 103) underwent cholecystectomy and had ACS, whereas 2.5% (n = 254) did not undergo cholecystectomy but developed ACS. Regarding chronic conditions, 27.4% (n = 17,610), 43.0% (n = 27,667), and 35.3% (n = 22,748) of patients had diabetes, hypertension, and dyslipidemia, respectively, in whom the incidence rates of ACS were 4.0% (n = 702), 4.0% (n = 1109), and 3.8% (n = 863), respectively (Table 1).

**Figure 1:**
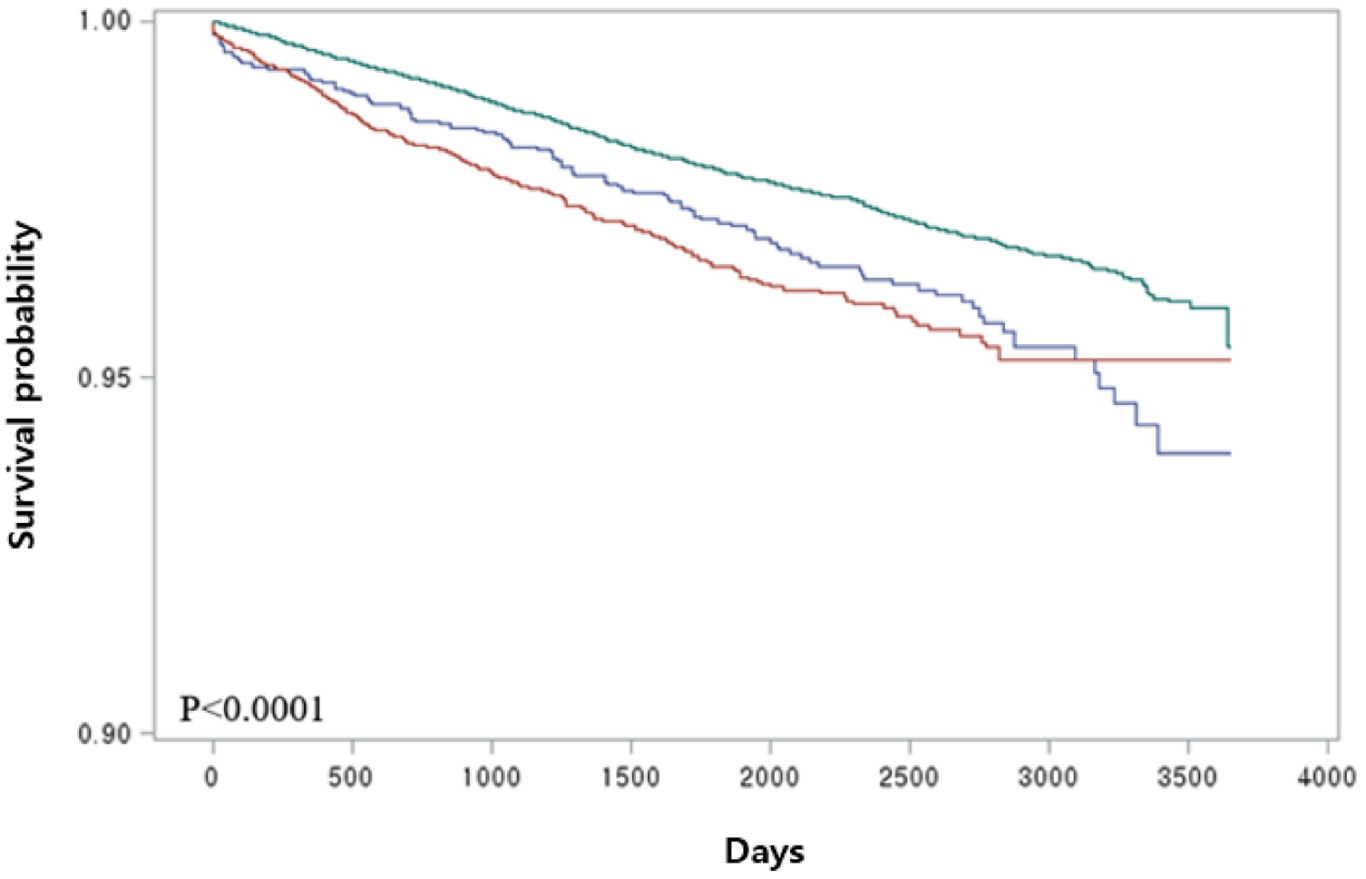
Risk of acute coronary syndrome and gallbladder stones (GS) Green: control group; Blue: GS with cholecystectomy; Red: GS without cholecystectomy.

Kaplan–Meier analysis showed that the GS group was at a higher risk of developing ACS regardless of cholecystectomy (Fig. 1**,** *P* < 0.0001). Table 2 shows the results of the Cox proportional hazard model using HRs between the variables and ACS. The GS group exhibited a higher risk of developing ACS than the control group (HR:1.30, 95% CI 1.15–1.47, *P* < 0.0001). Individuals with GS had a higher risk of ACS (HR: 1.30, 95% CI 1.15–1.47, *P* < 0.0001) compared to the control group. Regarding cholecystectomy in the GS group, individuals who did not undergo cholecystectomy had a statistically higher risk of ACS development (HR: 1.35, 95% CI 1.17–1.55, *P* < 0.0001) compared to the control group, while the risk did not significantly differ after cholecystectomy compared to the control group (HR: 1.21, 95% CI 0.98–1.48, *P* = 0.0755).

**Table 2:** Risk factors related to acute coronary syndrome.

	Acute coronary syndrome		
	HR	95% CI	*p*-value
Gallbladder stone			
Yes	1.30	(1.15–1.47)	< .0001
Cholecystectomy Yes	1.21	(0.98–1.48)	0.0755
Cholecystectomy No	1.35	(1.17–1.55)	< .0001
No	1.00	–	
Age group (years)			
20–29	0.59	(0.23–1.52)	0.2742
30–39	1.00	–	
40–49	1.30	(0.88–1.93)	0.1943
50–59	1.95	(1.35–2.83)	0.0004
60–69	2.63	(1.82–3.80)	< .0001
70–79	3.00	(2.06–4.37)	< .0001
80–89	2.85	(1.86–4.36)	< .0001
Sex			
Male	1.37	(1.22–1.55)	< .0001
Female	1.00	–	
Regions			
Capital area	1.00	–	
Metropolitan area	1.21	(1.05–1.39)	0.0077
Rural area	1.18	(1.04–1.35)	0.0104
Income level			
Low	1.00	–	
Medium	0.97	(0.82–1.14)	0.7063
High	1.03	(0.88–1.21)	0.6968
Occupational status			
Working	1.00	(0.88–1.14)	0.9767
Not working	1.00	–	
Disability			
Yes	1.15	(0.98–1.36)	0.0864
No	1.00	–	
Diabetes			
Yes	1.29	(1.15–1.45)	< .0001
No	1.00	–	
Hypertension			
Yes	3.74	(3.14–4.47)	< .0001
No	1.00	–	
Dyslipidemia			
Yes	1.98	(1.75–2.24)	< .0001
No	1.00	–	
CCI†			
0	1.00	–	
1	1.70	(1.19–2.44)	0.0039
2	1.61	(1.13–2.28)	0.008
3	2.31	(1.69–3.16)	< .0001
Cohort entry year			
2004	1.00	–	
2005	1.03	(0.84–1.26)	0.7954
2006	1.01	(0.82–1.24)	0.9484
2007	0.87	(0.70–1.09)	0.2201
2008	1.01	(0.81–1.26)	0.9298
2009	0.88	(0.69–1.12)	0.311
2010	1.00	(0.78–1.28)	0.9816
2011	0.86	(0.65–1.13)	0.2748
2012	0.92	(0.67–1.25)	0.5815
2013	1.37	(0.92–2.05)	0.1208

^**†**^Charlson Comorbidity Index.

We further investigated the association between GS and ACS according to diabetes, hypertension, and dyslipidemia (Tables 2 and 3). Diabetes (HR:1.29, 95% CI 1.15–1.45, *P* < 0.0001), hypertension (HR:3.74, 95% CI 3.14–4.47, *P* < 0.0001), and dyslipidemia (HR:1.98, 95% CI 1.75–2.24, *P* < 0.0001) (Table 2) were all associated with a higher risk of ACS development in this study. GS was also associated with the development of ACS in patients with metabolic diseases. The presence of GS increased the risk of ACS in those with metabolic diseases (diabetes HR:1.38, 95% CI 1.18–1.63, *P* < 0.0001; hypertension HR:1.23, 95% CI 1.08–1.41, *P* = 0.0023; dyslipidemia HR:1.22, 95% CI 1.05–1.41, *P* = 0.0106) (Table 3). The risk of developing ACS increased with the presence of diabetes, hypertension, and dyslipidemia in patients with GS compared to those without GS (HR:1.29, 95% CI 1.14 – 1.47, *P* < 0.0001).

**Table 3:** Additional risk factors of acute coronary syndrome associated with gallbladder stones (GS).

	Stone (−)	Acute Coronary Syndrome								
		Stone ( +)			Cholecystectomy					
					Yes			No		
		HR	95% CI	*P*-value	HR	95% CI	*P*-value	HR	95% CI	*P*-value
Diabetes										
Yes	1.00	1.38	(1.18–1.63)	< .0001	1.21	(0.93–1.58)	0.1631	1.44	(1.20–1.73)	< .0001
No	1.00	1.25	(1.03–1.52)	0.0234	1.19	(0.86–1.66)	0.2928	1.22	(0.97–1.52)	0.0892
Hypertension										
Yes	1.00	1.23	(1.08–1.41)	0.0023	1.09	(0.87–1.38)	0.4395	1.26	(1.08–1.47)	0.0031
No	1.00	2.12	(1.54–2.93)	< .0001	2.13	(1.29–3.52)	0.0030	2.00	(1.39–2.89)	0.0002
Dyslipidemia										
Yes	1.00	1.22	(1.05–1.41)	0.0106	1.00	(0.77–1.30)	0.9766	1.31	(1.10–1.55)	0.0019
No	1.00	1.55	(1.25–1.93)	< .0001	1.78	(1.27–2.50)	0.0008	1.44	(1.12–1.86)	0.0051
All three diagnosis										
Yes	1.00	1.29	(1.14–1.47)	< .0001	1.15	(0.93–1.43)	0.1924	1.30	(1.13–1.50)	0.0004
No	1.00	2.66	(1.54–4.60)	0.0005	2.93	(1.27–6.76)	0.0116	2.54	(1.35–4.76)	0.0037

HR, hazards ratio; CI, confidence interval.

The gallstone group was subdivided into two groups according to cholecystectomy. The impact of cholecystectomy on ACS differed according to the type of metabolic disease. In patients with diabetes, hypertension, or dyslipidemia, the gallstone groups who did not undergo cholecystectomy had a higher risk of ACS development than those who underwent cholecystectomy (diabetes HR:1.44, 95% CI 1.20–1.73, *P* < 0.0001; hypertension HR:1.26, 95% CI 1.08–1.47, *P* = 0.0031; dyslipidemia HR:1.31, 95% CI 1.10–1.55, *P* = 0.0019) (Table 3). Among the gallstone group diagnosed with any of the three diseases, those who did not undergo cholecystectomy showed a higher risk of ACS development than those who underwent cholecystectomy (HR,1.30; 95% CI 1.13–1.50; *P* = 0.0004). In contrast, in patients without the three diseases, cholecystectomy was associated with a higher risk of ACS development (HR:2.93, 95% CI 1.27–6.76, *P* = 0.0116).

## Discussion

Previous studies have reported that the presence of GS is associated with an increased risk of ACS. Our results verified this and additionally showed that patients without cholecystectomy for GS are associated with an increased risk of ACS compared to the control group. Interestingly, the effects of cholecystectomy for GS on the risk of ACS differed depending on the underlying metabolic disease. When cholecystectomy was performed on patients with GS with an underlying metabolic disease, such as hypertension, diabetes, or hyperlipidemia, the risk of ACS was not significantly different compared to those without GS, but this trend was reversed in the GS group without underlying metabolic disease. In patients without all three diseases (diabetes, hypertension, and dyslipidemia), cholecystectomy was still associated with an increased risk of ACS than the control group. However, when the diseases were considered individually, the presence or absence of diabetes alone was not associated with an increased risk of ACS after cholecystectomy. These findings imply that when cholecystectomy is planned for GS, underlying metabolic diseases should be considered to reduce the risk of ACS.

Previous studies and our results have shown a significantly increased risk of ACS among patients with GS^4,5,7,12^. Patients with GS frequently have risk factors for ACS, which could explain the increased risk of ACS. Our subgroup analysis showed that GS was associated with an even higher risk of ACS in patients with diabetes, hyperlipidemia, and hypertension. Therefore, patients with gallstones were at increased risk of developing ACS not simply because they already had other risk factors, but because the presence of GS itself posed a risk.

Age, obesity, body mass index, low serum high-density lipoprotein cholesterol levels, diabetes mellitus, inadequate physical inactivity, and excessive alcohol use have all been identified as ACS risk factors^7^. These are also shared risk factors for GS, which could explain the increased risk of ACS in the GS group^13^. The formation of gallstones is thought to be caused primarily by biliary cholesterol supersaturation caused by metabolic abnormalities in the liver and the subsequent physico-chemical imbalance of cholesterol solubility in bile^4,14,15^. The term “ACS” refers to a group of conditions characterized by a sudden decrease in blood flow to the heart, such as unstable angina, non-ST elevation myocardial infarction, and ST elevation myocardial infarction.

The association between GS and ACS is easily understood but is unpredictable with cholecystectomy. The previous studies have reported varied effects of cholecystectomy on the risk of ACS. Chen et al. investigated the effect of cholecystectomy on the subsequent risk of ACS in patients with GS^10^. The cumulative incidence of ACS was lower in patients with GS who underwent cholecystectomy than in those who did not. In contrast, Norberto et al. analyzed the relationship between cholecystectomy for GS and risk factors for ACS. Multivariate analysis revealed that the cholecystectomy group had a higher incidence of metabolic risk variables for ACS than the control group. Patients who underwent cholecystectomy exhibited a higher incidence of risk factors for ACS regardless of age, sex, or body mass index^9^. In terms of patients’ baseline characteristics, the study by Chen et al. had a higher proportion of patients with metabolic illnesses than the study by Norberto et al. This suggests that the effect of cholecystectomy on the risk of ACS may differ depending on the underlying presence of metabolic diseases. Our results prove that the above-mentioned hypothesis is most likely to be true. In patients with metabolic diseases, not undergoing cholecystectomy was associated with a higher risk of ACS than the control group, but the risk did not significantly differ after cholecystectomy compared to the control group. Among patients without metabolic disorders, cholecystectomy was still associated with increased ACS risk in the GS group compared to the control group. These findings should be verified in future studies.

Although the treatment of asymptomatic gallstone patients is not generally recommended, current treatment strategies for symptomatic gallstone patients require surgical treatment. Following cholecystectomy, the gallbladder reservoir decreases, resulting in the continuous release of hepatic bile into the duodenum lumen^16^. As a result, the bile acid pool circulates more quickly, exposing enterohepatic organs and, eventually, peripheral tissues to a larger flow of bile acids during the diurnal cycle fasting periods^17^. Few studies have investigated the serum lipid profiles of individuals who underwent laparoscopic cholecystectomy. They showed that lipid and bile acid metabolisms are functionally linked^18^. There have been several hypotheses suggesting that cholecystectomy affects the lipid profile, which in turn affects the risk of ACS^19,20^. However, the results of previous studies cannot fully explain our findings, therefore, more research on the mechanism is required.

On the other hand, we also regard the mechanistic link between gallstones and cardiovascular disease that is associated with the gut microbiome. Patients with gallstones exhibit a distorted secretion of bile acids that play a crucial role in regulating gut microbiota^21^. Dysbiosis of gut microbiota may influence various host functions associated with cardiovascular risk^22–24^. Previous studies have reported that metabolites produced by gut microbiota, such as trimethylamine N-oxide and L-carnitine, inhibit bile acid transporters and promote atherosclerosis and cardiovascular risk^25,26^. Proteobacteria has been found to be linked to a wide range of metabolic disturbances, including an increased risk of obesity and cardiovascular disease^27,28^. Cholecystectomy alters bile flow to the intestine and can, therefore, modify the bidirectional interactions between bile acids and the intestinal microbiota^29^.

This study had several limitations First, the analysis was based on health insurance claim data reported with ICD-10 diagnostic codes; therefore, potential covariates, such as health behavior factors, drug prescription or laboratory test results were not included in this study. Due to a lack of clinical and laboratory data, there are still problems with diagnosis accuracy and failure to account for risk variables for gallstone disease. We adjusted all data for CCI, which provides information on the severity of an individual’s health conditions. In the future, we plan to investigate the relationship between metabolic disease and cholecystectomy using the same data from the Korea National Health and Nutrition Examination Survey. Second, the characteristics, including the number and size of gallstones, which are important factors related to gallstone surgery and prognosis, could not be investigated in this study. This aspect should be recognized as a limitation of epidemiological studies based on health insurance claims data. This study aimed to provide comprehensive information using a large-scale epidemiological study, and it was the largest large-scale study to examine the effect of cholecystectomy on ACS in patients with gallstones to date.

In conclusion, GS was associated with an increased risk of ACS. When cholecystectomy was performed for GS in patients with underlying metabolic diseases, the risk of ACS was not significantly different from that in patients without GS. When cholecystectomy is planned, its effect on the risk of ACS should be considered. Further validation in a large prospective cohort study and elucidation of the underlying mechanisms are needed.

## Methods

In this study, we used data from the Korean National Health Insurance Service National Sample Cohort (NHIS-NSC). The NHIS is the universal health insurance service in Korea, under which all citizens are enrolled, as such, all Korean citizens’ claims data are included in the NHIS database. The NHIS-NSC data contains 2.2% of the entire Korean population, selected using a systematic stratified random sampling method. Data were collected over 11 years from 2002 to 2013. The International Statistical Classification of Diseases and Related Health Problems, 10th revision (ICD-10), was used for the health insurance claim data.

The final study population of 64,370 individuals was selected after applying 2 years of wash-out periods to detect newly diagnosed patients with ACS or GS. Of the total study population, 13,069 patients with GS were stratified to the gallstone group, while 51,301 individuals without GS were designated as the control group. To establish the case and control groups, a 1:3 propensity score matching of age, sex, and year was performed. The matched individuals shared the same index date as the case group, taking the diagnosis date as their follow-up start date.

GS and ACS were defined using the ICD-10 codes. K80.0, K80.1, and K80.2 defined GS, while I20.0 (unstable angina) and I21 (AMI) defined ASC. Once an individual was diagnosed with one of these ICD codes, they were categorized as patients. Patients with GS were further subdivided into those who underwent cholecystectomy and those who did not. As covariate variables, sex, age, region, income level, occupational status, disability, diabetes, hypertension, dyslipidemia, Charlson comorbidity index (CCI), and cohort entry year were examined. The age group was divided into 10-year intervals from the 20 s to the 80 s. None of the individuals were aged over 89 years. The regions were divided into three groups: the capital area (Seoul), metropolitan area (Busan, Daegu, Daejeon, Gwangju, Incheon, and Ulsan), and rural areas (Gyeonggi, Gangwon, Chungcheongbuk, Chungcheongnam, Jeollabuk, Jeollanam, Gyeongsangbuk, Gyeongsangnam, and Jeju). Diabetes (E10, E11, E12, E13, and E14), hypertension (I10, I11, I12, I13, I14, and I15), and dyslipidemia (E78) were defined using the ICD-10 codes.

Descriptive statistics for each variable of ACS are presented. Chi-square tests were used to test the differences in variables and incidence of ACS at baseline. Kaplan–Meier curves and log-rank test were applied to investigate the association between GS and ACS, along with survival time. The multivariate Cox proportional hazard model was employed to estimate the adjusted hazard ratios (HRs) and 95% confidence intervals (CI). The analyses investigated the associations between variables and ACS, considering survival time. All analyses were performed using SAS software (version 9.4; SAS Institute, Cary, North Carolina, USA).

### Ethical approval and informed consent

The study used de-identified secondary health insurance claim data collected and distributed by NHIS under the government Ministry of Health and Welfare. As the data is available upon request to NHIS (www.nhis.or.kr), the study received a waiver of Ethical approval from Yonsei University Health System, Severance Hospital, Institutional Review Board (Wavier approval number: 4-2021-1375). Moreover, the informed consent was waived as the data is provided by NHIS. The waiver was approved based as Enforcement Rules Of The Bioethics and Safety Act, Republic of Korea [Article 15 (Deliberation on Human Subjects Research Projects)(1) A person who intends to conduct a human subjects research project shall prepare a research plan and submit it for deliberation by the competent IRB before commencing such human subjects research project. (2) Notwithstanding paragraph (1), a research project may be exempted from deliberation by the competent IRB, if a risk to human subjects of research and the general public is insignificant and the research project meets the standards prescribed by Ordinance of the Ministry of Health and Welfare after deliberation by the National Committee].

## Supplementary Information

Supplementary Table S1.

## Data Availability

Deidentified individual participant data are available upon request to Korean National Health Insurance Sharing Service at https://nhiss.nhis.or.kr/.

## Abbreviations

ACS: Acute coronary syndrome
CCI: Charlson comorbidity index
GS: Gall stone
HR: Hazards ratio
CI: Confidence interval
NHIS-NSC: Korean National Health Insurance Service National Sample Cohort

## References

[1] Froutan Y (2015). Gallstone disease founded by ultrasonography in functional dyspepsia: prevalence and associated factors. Int. J. Clin. Exp. Med..

[2] Shaffer EA (2006). Epidemiology of gallbladder stone disease. Best Pract. Res. Clin. Gastroenterol..

[3] Housset C, Chretien Y, Debray D, Chignard N (2016). Functions of the gallbladder. Compr. Physiol..

[4] Fairfield CJ, Wigmore SJ, Harrison EM (2019). Gallstone disease and the risk of cardiovascular disease. Sci. Rep..

[5] Olaiya MT, Chiou HY, Jeng JS, Lien LM, Hsieh FI (2013). Significantly increased risk of cardiovascular disease among patients with gallstone disease: a population-based cohort study. Plos One.

[6] Zheng Y (2016). Gallstones and risk of coronary heart disease: prospective analysis of 270 000 men and women from 3 US cohorts and meta-analysis. Arterioscler. Thromb. Vasc. Biol..

[7] Fan LL, Chen BH, Dai ZJ (2017). The relation between gallstone disease and cardiovascular disease. Sci. Rep..

[8] Lozano R (2012). Global and regional mortality from 235 causes of death for 20 age groups in 1990 and 2010: a systematic analysis for the Global Burden of Disease Study 2010. The Lancet.

[9] Chavez-Tapia NC (2012). Association between cholecystectomy for gallstone disease and risk factors for cardiovascular disease. Ann. Hepatol..

[10] Chen CH, Lin CL, Kao CH (2021). The effect of cholecystectomy on the risk of acute myocardial infarction in patients with gallbladder stones. Postgrad. Med..

[11] Chen YS, Wu SD, Tian Y (2018). Cholecystectomy as a risk factor of metabolic syndrome: from epidemiologic clues to biochemical mechanisms. Lab. Invest..

[12] Fairfield C, Wigmore S, Harrison E (2018). Gallstone disease and the risk of cardiovascular disease: systematic review and meta-analysis of observational studies. Brit. J. Surg..

[13] Huang JJ (2020). The association between gallstone disease (GSD) and the incidence of prediabetes and type 2 diabetes mellitus (type 2 DM): a prospective cohort study. Int. J. Diabetes Dev. C.

[14] Lammert F (2016). Gallstones. Nat. Rev. Dis. Primers.

[15] Portincasa P, Moschetta A, Palasciano G (2006). Cholesterol gallstone disease. Lancet.

[16] Amigo L (2011). Cholecystectomy increases hepatic triglyceride content and very-low-density lipoproteins production in mice. Liver Int..

[17] Hylemon PB (2009). Bile acids as regulatory molecules. J. Lipid Res..

[18] Kuipers F, Bloks VW, Groen AK (2014). Beyond intestinal soap–bile acids in metabolic control. Nat. Rev. Endocrinol..

[19] Sergeev I, Keren N, Naftali T, Konikoff FM (2020). Cholecystectomy and biliary sphincterotomy increase fecal bile loss and improve lipid profile in dyslipidemia. Dig. Dis. Sci..

[20] Krondl A, Vavrinkova H, Michalec C (1964). effect of cholecystectomy on the role of the gall bladder in fat absorption. Gut.

[21] Berr F, Pratschke E, Fischer S, Paumgartner G (1992). Disorders of bile acid metabolism in cholesterol gallstone disease. J. Clin. Invest..

[22] Wang Z (2011). Gut flora metabolism of phosphatidylcholine promotes cardiovascular disease. Nature.

[23] Vinjé S, Stroes E, Nieuwdorp M, Hazen SL (2014). The gut microbiome as novel cardio-metabolic target: the time has come!. Eur. Heart J..

[24] Tang WH (2013). Intestinal microbial metabolism of phosphatidylcholine and cardiovascular risk. N. Engl. J. Med..

[25] Koeth RA (2013). Intestinal microbiota metabolism of L-carnitine, a nutrient in red meat, promotes atherosclerosis. Nat. Med..

[26] Charach G (2011). The association of bile acid excretion and atherosclerotic coronary artery disease. Therap. Adv. Gastroenterol..

[27] Wu T (2013). Gut microbiota dysbiosis and bacterial community assembly associated with cholesterol gallstones in large-scale study. BMC Genom..

[28] Carey MC, Mazer NA (1984). Biliary lipid secretion in health and in cholesterol gallstone disease. Hepatology.

[29] Keren N (2015). Interactions between the intestinal microbiota and bile acids in gallstones patients. Environ. Microbiol. Rep..

