# Supplementary material for: Impact of cholecystectomy on acute coronary syndrome according to metabolic condition: a nationwide population-based cohort study
Source: Sci Rep. 2023 May 5;13:7300. doi: 10.1038/s41598-023-33440-4 (PMC10163235; doi:10.1038/s41598-023-33440-4)
Supplement: Supplementary file 1 — Supplementary Table S1. [file 41598_2023_33440_MOESM1_ESM.docx]

Supplementary Table 1. Study population distribution by gallbladder stone

|  | | **Gallbladder stone** | | | | | | | | | |
| --- | --- | --- | --- | --- | --- | --- | --- | --- | --- | --- | --- |
|  |  | **Total** | **Yes** | | | | | | **No** | | **P-value** |
|  |  |  | **Total** | **%** | **Cholecystectomy Yes** | | **Cholecystectomy No** | |  |  |  |
|  |  |  |  |  | **N** | **%** | **N** | **%** | **N** | **%** |  |
| **Total** | | **64370** | **13069** |  | **2706** | 4.2 | **10363** | 16.1 | **51301** | 79.7 | <.0001 |
| **Age group** | |  |  |  |  |  |  |  |  |  | <.0001 |
|  | 20~29 | 3100 | 622 | 4.8 | 144 | 5.3 | 478 | 4.6 | 2478 | 4.8 |  |
|  | 30~39 | 9035 | 1806 | 13.8 | 469 | 17.3 | 1337 | 12.9 | 7229 | 14.1 |  |
|  | 40~49 | 12628 | 2530 | 19.4 | 553 | 20.4 | 1977 | 19.1 | 10098 | 19.7 |  |
|  | 50~59 | 14548 | 2927 | 22.4 | 589 | 21.8 | 2338 | 22.6 | 11621 | 22.7 |  |
|  | 60~69 | 12484 | 2551 | 19.5 | 528 | 19.5 | 2023 | 19.5 | 9933 | 19.4 |  |
|  | 70~79 | 9341 | 1944 | 14.9 | 346 | 12.8 | 1598 | 15.4 | 7397 | 14.4 |  |
|  | 80~89 | 3234 | 689 | 5.3 | 77 | 2.8 | 612 | 5.9 | 2545 | 5.0 |  |
| **Sex** | |  |  |  |  |  |  |  |  |  | 0.6118 |
|  | Male | 30778 | 6286 | 48.1 | 1286 | 47.5 | 5000 | 48.2 | 24492 | 47.7 |  |
|  | Female | 33592 | 6783 | 51.9 | 1420 | 52.5 | 5363 | 51.8 | 26809 | 52.3 |  |
| **Regions** | |  |  |  |  |  |  |  |  |  | 0.0072 |
|  | Capital area | 26850 | 5238 | 40.1 | 1179 | 43.6 | 4059 | 39.2 | 21612 | 42.1 |  |
|  | Metropolitan area | 16437 | 3478 | 26.6 | 674 | 24.9 | 2804 | 27.1 | 12959 | 25.3 |  |
|  | Rural area | 21083 | 4353 | 33.3 | 853 | 31.5 | 3500 | 33.8 | 16730 | 32.6 |  |
| **Income level** | |  |  |  |  |  |  |  |  |  | <.0001 |
|  | Low | 11593 | 2052 | 15.7 | 325 | 12.0 | 1727 | 16.7 | 9541 | 18.6 |  |
|  | Medium | 26302 | 5262 | 40.3 | 1136 | 42.0 | 4126 | 39.8 | 21040 | 41.0 |  |
|  | High | 26475 | 5755 | 44.0 | 1245 | 46.0 | 4510 | 43.5 | 20720 | 40.4 |  |
| **Occupational status** | |  |  |  |  |  |  |  |  |  | <.0001 |
|  | Working | 31593 | 6229 | 47.7 | 1196 | 44.2 | 5033 | 48.6 | 25364 | 49.4 |  |
|  | Not working | 32777 | 6840 | 52.3 | 1510 | 55.8 | 5330 | 51.4 | 25937 | 50.6 |  |
| **Disability** | |  |  |  |  |  |  |  |  |  | <.0001 |
|  | Yes | 5528 | 1267 | 9.7 | 219 | 8.1 | 1048 | 10.1 | 4261 | 8.3 |  |
|  | No | 58842 | 11802 | 90.3 | 2487 | 91.9 | 9315 | 89.9 | 47040 | 91.7 |  |
| **Diabetes** | |  |  |  |  |  |  |  |  |  | <.0001 |
|  | Yes | 17610 | 4515 | **34.5** | 973 | **36.0** | 3542 | **34.2** | 13095 | **25.5** |  |
|  | No | 46760 | 8554 | 65.5 | 1733 | 64.0 | 6821 | 65.8 | 38206 | 74.5 |  |
| **Hypertension** | |  |  |  |  |  |  |  |  |  | <.0001 |
|  | Yes | 27667 | 6303 | **48.2** | 1344 | **49.7** | 4959 | **47.9** | 21364 | **41.6** |  |
|  | No | 36703 | 6766 | 51.8 | 1362 | 50.3 | 5404 | 52.1 | 29937 | 58.4 |  |
| **Dyslipidemia** | |  |  |  |  |  |  |  |  |  | <.0001 |
|  | Yes | 22748 | 5607 | **42.9** | 1182 | **43.7** | 4425 | **42.7** | 17140 | **33.4** |  |
|  | No | 41622 | 7461 | 57.1 | 1523 | 56.3 | 5938 | 57.3 | 34161 | 66.6 |  |
| **CCI†** |  |  |  |  |  |  |  |  |  |  | <.0001 |
|  | 0 | 10452 | 962 | 7.4 | 183 | 6.8 | 779 | 7.5 | 9490 | 18.5 |  |
|  | 1 | 9428 | 1274 | 9.7 | 211 | 7.8 | 1063 | 10.3 | 8154 | 15.9 |  |
|  | 2 | 9978 | 1912 | 14.6 | 420 | 15.5 | 1492 | 14.4 | 8066 | 15.7 |  |
|  | 3 | 34512 | 8921 | 68.3 | 1892 | 69.9 | 7029 | 67.8 | 25591 | 49.9 |  |
| **Cohort entry year** | |  |  |  |  |  |  |  |  |  | <.0001 |
|  | 2004 | 4902 | 980 | 7.5 | 401 | 14.8 | 579 | 5.6 | 3922 | 7.6 |  |
|  | 2005 | 5271 | 1056 | 8.1 | 478 | 17.7 | 578 | 5.6 | 4215 | 8.2 |  |
|  | 2006 | 5590 | 1127 | 8.6 | 506 | 18.7 | 621 | 6.0 | 4463 | 8.7 |  |
|  | 2007 | 6065 | 1219 | 9.3 | 469 | 17.3 | 750 | 7.2 | 4846 | 9.4 |  |
|  | 2008 | 6346 | 1286 | 9.8 | 498 | 18.4 | 788 | 7.6 | 5060 | 9.9 |  |
|  | 2009 | 6141 | 1250 | 9.6 | 221 | 8.2 | 1029 | 9.9 | 4891 | 9.5 |  |
|  | 2010 | 6640 | 1350 | 10.3 | 18 | 0.7 | 1332 | 12.9 | 5290 | 10.3 |  |
|  | 2011 | 7741 | 1572 | 12.0 | 17 | 0.6 | 1555 | 15.0 | 6169 | 12.0 |  |
|  | 2012 | 8303 | 1700 | 13.0 | 36 | 1.3 | 1664 | 16.1 | 6603 | 12.9 |  |
|  | 2013 | 7371 | 1529 | 11.7 | 62 | 2.3 | 1467 | 14.2 | 5842 | 11.4 |  |
